# Neutral diagnosis: An innovative concept for medical device clinical trials

**DOI:** 10.1016/j.conctc.2019.100436

**Published:** 2019-08-21

**Authors:** Bo Zhang, Shangyuan Ye, Sravya B. Shankara, Hui Zhang, Qingfeng Zheng

**Affiliations:** a Department of Population and Quantitative Health Sciences, University of Massachusetts Medical School, Worcester, MA, 01605, USA; b Division of Biostatistics, Department of Preventive Medicine, Feinberg School of Medicine, Northwestern University, Chicago, IL, 60611, USA; c Department of Thoracic Surgery, National Cancer Center/ National Clinical Research Center for Cancer/ Cancer Hospital, Chinese Academy of Medical Sciences and Peking Union Medical College, Beijing, 100021, China

**Keywords:** Diagnostic devices, Drug-device combination product, Cancer, Neutral diagnosis, Surgical resection

## Abstract

Study design and statistical analysis are crucial in pivotal clinical trials to evaluate the effectiveness and safety of new medical devices under investigation. In recent years, innovative intraoperative *in vivo* breast tumor diagnostic devices have been proposed to improve the accuracy and surgical outcomes of breast tumor patients undergoing resection. Although such technologies are promising, investigators need to obtain statistical evidence for the effectiveness and safety of these devices by conducting valid clinical trials. However, the study design and statistical analysis for these clinical trials are complicated. While these trials are designed to provide real-time intraoperative diagnosis of cancerous tissue, they also have clear therapeutic objectives to lower the reoperation rate of breast cancer surgery. This research article introduces the new concept of neutral diagnosis (ND), and the ND clinical trial design as an innovative study design to evaluate the effectiveness and safety of diagnostic devices with direct therapeutic purposes. A joint modeling approach is adopted to make inferences on the effectiveness and safety of these devices for non-neutral diagnosis (non-ND) clinical trials. Simulation studies were conducted to show the efficiency of the ND trials and strength of the joint modeling approach in the non-ND clinical trials. An example on a diagnostic medical device that provides real-time, intraoperative diagnosis of breast cancer tumor tissues during breast cancer surgeries is comprehensively discussed and analyzed.

## 1 Introduction

Study design and statistical analysis are crucial in the pivotal clinical trials evaluating the effectiveness and safety of investigational medical devices. Study design and statistical analysis strategies have proven to be challenging for the devices that are based on recently developed breakthrough technologies with complex diagnostic and therapeutic features. In recent years, the Center for Devices and Radiological Health (CDRH) under the U.S. Food and Drug Administration (FDA) has received several applications for innovative intraoperative *in vivo* diagnostic devices for breast tumors, which are aimed at improving the accuracy and outcomes of surgical resection in breast tumor patients.

Performance of the real-time intraoperative margin-assessment device “MarginProbe” [1,2] in breast-conserving surgery has been studied in several clinical studies. Thill ([3]) compared and summarized the results from three major studies and introduced some improved features in alternative devices. The MAST study [2] was the first randomized clinical trial (RCT) studying the performance of the MarginProbe device. Patients were randomized to two groups, receiving either the standard of care (SOC) or the SOC with the aid of the new device. The results from this trial showed that the group receiving SOC with the new device had a significantly higher correct intraoperative surgical resection rate and a lower re-excision rate than the SOC group. Two other studies, the US Pivotal trial [4,5] and the German multicenter study [6,7], were also conducted. The US Pivotal trial was the largest among the three clinical trials and included 596 patients from 21 sites in the US and Israel. Both the US Pivotal trial and the German multicenter study concluded that the MarginProbe significantly reduced the re-excision rate and had no negative effects on the cosmetic outcomes of the patients. Other clinical trials on the MarginProbe are reported in Sebastian et al. [8] and Blohmer et al. [9]. These studies concluded that the assessment of intraoperative margins provided by the MarginProbe during breast-conserving surgery resulted in a reduction of re-excision rates. Reviews within Pappo et al. [10] and Thill et al. [11] included detailed device description on the MarginProbe and other similar devices.

The goal of our current research is to resolve the study design and data analysis issues in the clinical trials for the investigational medical devices described above. Our study introduces a new concept named “neutral diagnosis” (ND) and the neutral diagnosis (ND) design, an innovative study design to evaluate effectiveness and safety of the diagnostic devices with direct therapeutic applications. By adopting the novel ND design, investigators (or sponsors) can assess the real contribution of the investigational device relative to potential confounding factors. In addition, a joint modeling approach is presented to make inferences on the effectiveness and safety of such devices, when the ND design is not feasible or ethical. A synthetic clinical example is discussed to show the efficiency of ND clinical trials and the strength of the joint modeling approach in non-ND clinical trials.

## 2 A clinical trial from FDA regulatory practice: the MarginProbe system

### 2.1 The MarginProbe system

The novel MarginProbe system, manufactured by Dune Medical Devices, Inc., was approved by the FDA in December 2012 ([12]). The MarginProbe system includes a detachable single-use, single-patient component probe and a console with a user interface system that includes a display, audio components, and operation buttons ([12]) that use electromagnetic waves. The probe is sold separately and connected to the console via a single connector. The MarginProbe system probe is used to sample the entire surface of the specimen ([12]), and users are advised to take 5–8 measurements per margin surface. If even one of the readings is positive, the *ex vivo* lumpectomy margin should be considered positive, and appropriate surgical action (excision of the margin) should be taken. Dune Medical Devices, Inc., designed and conducted a pivotal prospective, multicenter, randomized (1:1), controlled, double-blinded clinical trial to establish reasonable assurance of safety and effectiveness for the MarginProbe system. Breast cancer patients were randomized to receive either a SOC lumpectomy or a SOC lumpectomy with the aid of an adjunctive MarginProbe device (SOC + device). Enrolled patients underwent resection of the main lumpectomy specimen. Resection of the main lumpectomy specimen, as well as lumpectomy cavity palpation and related re-excisions, were performed before randomizing the patients. Patients were then randomized to either the SOC or SOC + device arm intraoperatively, immediately after the main lumpectomy specimen was excised, oriented, center marked, palpated, and any additional palpation-based re-excision was performed. For patients in the SOC and SOC + device arms, lumpectomy specimens were analyzed by ultrasound or radiography after randomization and use of the device. Additional lumpectomy cavity re-excisions were taken appropriately, based on the imaging results of specimens (see Ref. [12] for the details of the study design). The primary effectiveness endpoint was set as the proportion of complete surgical re-excision (CSR) measured as all pathologically positive margins on the main specimen being intraoperatively re-excised or addressed. A re-excised or addressed margin does not mean that the final true outermost margin is pathologically negative for cancer. The results from this pivotal clinical trial indicated that the use of the MarginProbe system resulted in a reduction of the reoperation rate by 5%. The CSR rates for the SOC and SOC + device arms were 22.4% and 71.8%, (*p*-value < 0.0001), respectively when comparing differences between the two groups.

### 2.2 Regulatory tribulations and challenges

Although the FDA approved the MarginProbe system, there were serious flaws in the clinical study for its premarket approval application. First, the primary prognostic endpoint should have been the reoperation rate (i.e., the re-excision procedure rate) after the lumpectomy, instead of CSR. The CSR has obvious limitations as a primary effectiveness endpoint. With this primary endpoint, the investigator cannot detect whether a shaving during surgery was taken due to clinical suspicion, imaging, or another assessment versus a positive reading on the MarginProbe device. They also cannot determine whether the shaving was taken before randomization or after specimen imaging. Second, the study design for the MarginProbe system was biased in favor of the study arm. The study design allowed the surgeons for an additional option to take lumpectomy cavity shavings on the patients in the SOC + device arm. However, the reason for taking additional shavings of the lumpectomy cavity (e.g., surgeon suspicion, ultrasound imaging, radiographic imaging, or a positive MarginProbe device reading) was not documented during the trial. This made it difficult to determine whether observed results were due to the use of the MarginProbe device or confounding factors. Third, diagnostic endpoints were not included as one of the primary endpoints. Fourth, it was unclear how margin-level (or patient-level) sensitivity and specificity, which were reported as indicators of diagnostic performance, affected the reoperation rate.

These limitations commonly occur in clinical trials investigating diagnostic medical devices with direct therapeutic objectives. The goal of our current study is to resolve these issues and ensure that the new study design and data analysis approach can benefit future regulatory practice regarding such devices.

## 3 Neutral diagnosis: study design

In this section and the next section, we introduce a new concept, “neutral diagnosis” (ND). The study design and statistical analysis related to this concept are proposed and discussed. Sponsors can implement this new study design and statistical analysis approach to address the issues related to evaluating the effectiveness and safety of new investigational medical devices.

### 3.1 Neutral diagnosis: an innovative concept for medical device trials

Medical devices such as the MarginProbe system are characterized specifically by their dual functions in both therapeutics and diagnosis. These devices are designed to provide intraoperative, real-time diagnosis on cancerous tissue during lumpectomy (diagnostic function), but are primarily used to improve the surgical outcomes of breast cancer patients (therapeutic function). As such, the real-time diagnostic performance of these devices is expected to affect the surgical outcomes, which are evaluated by the reoperation rate. To thoroughly assess the effectiveness of such devices, we introduce a new concept, “neutral diagnosis.”

Definition 1 *(Neutral diagnosis): A diagnosis procedure is referred to as an ND procedure if the procedure uniformly provides a diagnosis with 50% sensitivity and 50% specificity, regardless of whether external and internal factors are specified. A diagnostic device is called an ND device if the device implements an ND procedure*.

The ND procedure or the ND device can be statistically interpreted as follows. Suppose $Y_{D}$ is the diagnostic outcome of a device, assuming binary values of either 1 representing a positive diagnostic result or 0 representing a negative diagnostic result. Let D denote the true status of disease or a gold standard. The fact that a diagnostic device is an ND device must imply that

$$P\left(Y_{D}=1\text{|}D=1\right)=P\left(Y_{D}=0\text{|}D=0\right)=0.5$$

Further, if $X=\left(X_{1},\;X_{2},\cdots ,X_{q}\right)$ denotes the vector of covariates, an ND device also satisfies

$$P\left(Y_{D}=1\text{|}D=1,\;X=x\right)=P\left(Y_{D}=0\text{|}D=0,\;X=x\right)=0.5,\;\text{for any }x\text{.}$$

Clearly, this covariate-adjusted ND is a sufficient but not necessary condition for the marginal ND. If the binary diagnostic outcome Y is decided by comparing the value of a continuous measurement $Y_{D}^{*}$ with a cut-off point c, such that $Y_{D}=1$ when $Y_{D}^{*}\geq c$ and $Y_{D}=0$ when $Y_{D}^{*}<c$, then an ND device also satisfies

$$P\left(Y_{D}^{*}\geq c\text{|}D=1,\;X=x\right)=P\left(Y_{D}^{*}<c\text{|}D=0,\;X=x\right)=0.5\;\text{for any }c\text{ and }x\text{.}$$

The diagnostic performance of an ND procedure or an ND device sets the bottom line for evaluating a practical diagnostic procedure or device. That is, any diagnostic procedure or device used in medical practice is expected to at least outperform the corresponding ND procedure or device. The ND devices are not practically useful. However, for medical device trials, the difference between the effectiveness of a new (proposed) device and the ND device is taken as a measure to evaluate the performance of the new device. The main objective of this research is to promote synthetic ND devices in pivotal clinical trials for medical devices and to encourage investigators and regulators to evaluate the performance of the proposed new device through the comparison between the investigational and ND devices.

### 3.2 Two-arm neutral diagnosis design

**Fig. 1:**
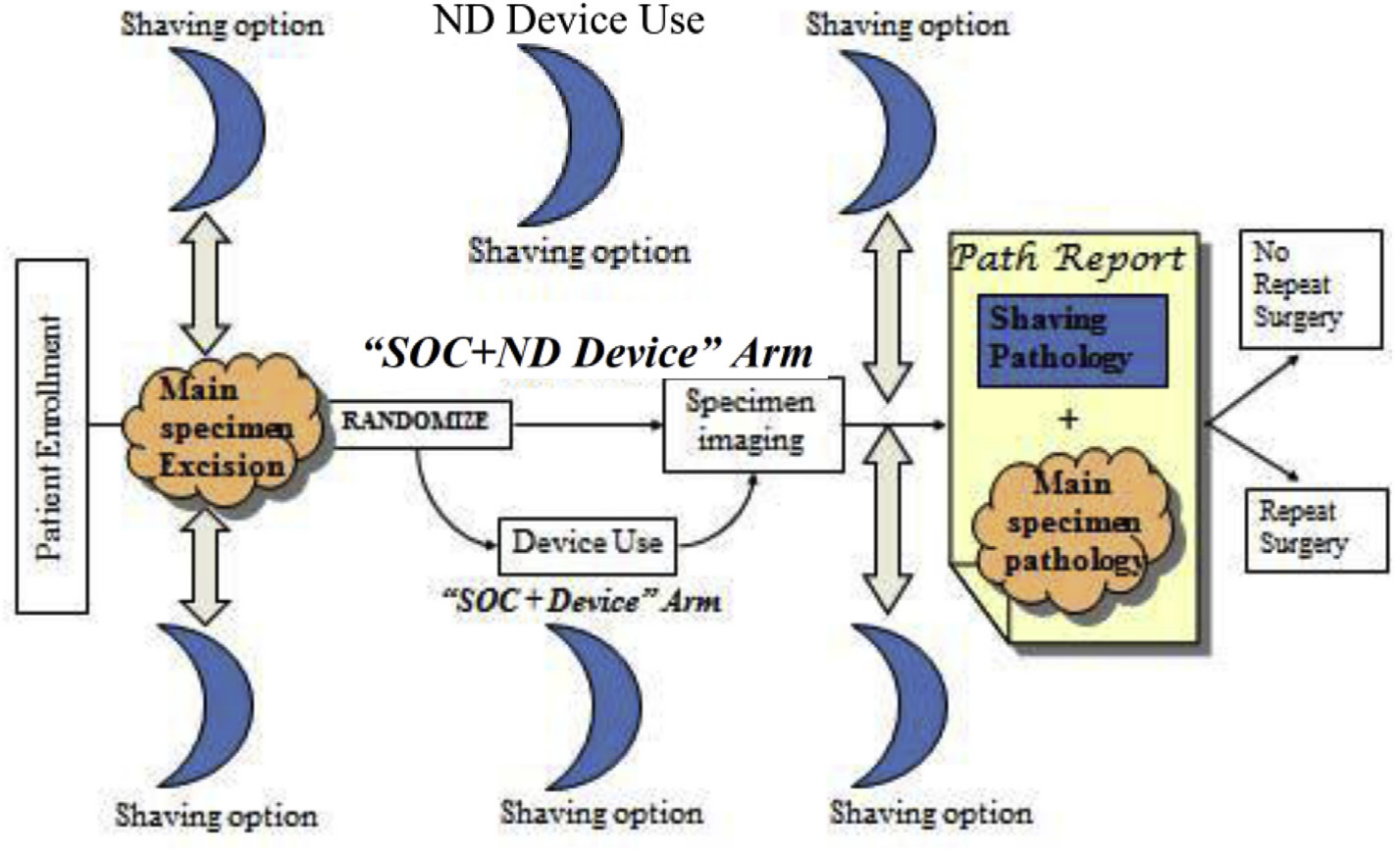
Two-arm neutral diagnosis design for evaluating the MarginProbe system that is modified from the clinical study reported in the Summary of Safety and Effectiveness Data of the MarginProbe System [12].

To overcome regulatory difficulties associated with study designs evaluating devices like the MarginProbe system, we propose a new study design for medical devices with both diagnostic and therapeutic functions. The clinical study for the MarginProbe system had two study arms: SOC and SOC + device (see Fig. 1). Here, we propose to design a clinical trial to compare one arm of the SOC + ND device with the arm of SOC + device. Fig. 1 shows the ND design for the MarginProbe system. The ND diagnostic device can be implemented by modifying the existing algorithm in a new device to achieve uniform ND. The use of an ND diagnostic device should be blinded to device users in the trials.

Definition 2 *(Two-Arm ND Study) A two-arm ND study for a medical device refers to a two-arm RCT, in which the first arm examines the performance of a new investigational device and the second arm implements a study protocol identical to that for the first arm, but with an ND device*.

In a clinical study examining the effectiveness of a medical device with both diagnostic and therapeutic functions, it is recommended to set up two co-primary endpoints: $Y_{D}$ is a binary diagnostic endpoint, and $Y_{T}$ is the prognostic endpoint affected by $Y_{D}$ and other observed or latent factors. Let W be the treatment assignment variable with W=1 representing the investigational device arm and W=0 representing the ND device arm. Then,

$${\text{Δ}}_{1}=E\left(Y_{T}\text{|}W=1\right)-E\left(Y_{T}\text{|}W=0\right)$$

is the average treatment effect of the investigational device relative to the ND device. We designate this effect as “treated-to-ND effect”. The advantage of conducting the ND study is that it can, by randomization, expel the effects of any observed or latent factors on the prognostic endpoint and can directly estimate the treated-to-ND effect for medical devices with both diagnostic and therapeutic functions.

### 3.3 Three-arm neutral diagnosis design

In the clinical study of the MarginProbe system and other similar medical devices, we can also design a clinical study to compare three arms: SOC only, SOC + ND device, and SOC + device.

Definition 3 *(Three-Arm ND Study) A three-arm ND study for a medical device refers to a three-arm RCT, in which the first arm examines the performance of a new investigational device, the second arm implements a study protocol identical to that for the first arm, but with a ND device, and the third arm uses a standard treatment procedure without using either the investigational or the ND device*.

Let W be the treatment assignment variable, with W=1 representing the investigational device arm, W=0 representing the ND device arm, and W=−1 representing the standard-care arm. Then,

$${\text{Δ}}_{2}=E\left(Y_{T}\text{|}W=0\right)-E\left(Y_{T}\text{|}W=-1\right)$$

is the average treatment effect of the ND device relative to the SOC (designated as the “ND-to-SOC effect”), and

$$\text{Δ}=E\left(Y_{T}\text{|}W=1\right)-E\left(Y_{T}\text{|}W=-1\right)$$

is the average treatment effect of the investigational device relative to the SOC (designated as the “treated-to-SOC effect”). Apparently, $\text{Δ}={\text{Δ}}_{1}+{\text{Δ}}_{2}$ holds.

## 4 Statistical analysis of non-neutral diagnosis studies

For the MarginProbe system, the sponsor implemented a non-neutral diagnosis (non-ND) study design, which is defined as follows.

Definition 4 *(Two-Arm Non-ND Study) A two-arm non-ND study for a medical device refers to a two-arm RCT, in which the first arm examines the performance of the new investigational device and the second arm treats patients by using a standard treatment procedure without using either the investigational or the ND device*.

Because a non-ND study includes only a SOC arm and a SOC + device arm, its disadvantage is that it can only assess the treated-to-SOC treatment effect, which may be largely dominated by the ND-to-SOC effect if the treated-to-ND effect is not at all significant. Note that the treated-to-SOC effect is the summation of the treated-to-ND effect and the ND-to-SOC effect. In this section, we propose an approach to estimate the treated-to-ND effect and the ND-to-SOC effect in a non-ND study. This enables sponsors to assess both these effects, even when the clinical trial is a non-ND study.

We only consider the data from the SOC + device arm in a non-ND study. Denote $Y_{Di}=\left(Y_{D_{1}i},\;Y_{D_{2}i},\;\cdots ,Y_{D_{J}i}\right)'$ as the J-length vector of multiple correlated diagnostic endpoints from the ith subject in the SOC + device arm for i=1,2,⋯,n. It is assumed that $Y_{Di}$, i=1,2,…,n, are independent and identically distributed. Each diagnostic endpoint, $Y_{D_{j}i}$, j=1,2,…J, takes the values of either 1, representing a positive diagnosis, or 0, representing a negative diagnosis. Let $D_{ji}$ be the true disease status or a gold standard of $Y_{D_{j}i}$. Let $X_{i}=\left(X_{1i},\;X_{2i},\cdots ,X_{pi}\right)$ denote the vector of baseline covariates. Consider joint models ([13,14]) of sensitivity and specificity:

P(YDji=1|Dji=1,Xi,bi)=Φ(μ1+Xi'β1+bi)

P(YDji=0|Dji=0,Xi,bi)=Φ(μ0+Xi'β0+λbi),

in which Φ is the probit link function, $\mu_{0},\;\;\mu_{1},\;\beta_{0}$ and $\beta_{1}$ are regression coefficients, and $b_{i}$ represents random effects that follow a normal distribution $b_{i}\sim N\left(0,\sigma^{2}\right).$This types of joint models are called the “shared-parameter models”. The unknown coefficients λ quantifies how much the sensitivity and the specificity are associate with each other. Denote $Y_{Ti}$ as the primary therapeutic (prognostic) endpoint for the ith subject in the SOC + device arm. Suppose that $Y_{T1},\;Y_{T2},\;\cdots ,\;Y_{Tn}$ independent and conditionally follow an exponential family of distribution

$$f\left(y_{Ti}\;\text{|}\;x_{i},\;\;b_{i}\right)=\exp\left(\left(y_{Ti}\vartheta_{i}-\psi \left(\vartheta_{i}\right)\right)/\varphi +\tau \left(y_{Ti},\;\varphi \right)\right)\text{,}$$

where ψ and τ are known functions, φ is the dispersion parameter, and $\vartheta_{i}$ is the parameter vector that depends on the covariate $X_{i}$ and the latent random effect $b_{i}$. To connect the therapeutic (prognostic) endpoint $Y_{Ti}$ with diagnostic endpoints $Y_{Di}$, we assume a generalized linear model for the conditional expectation:

E(YTi|Xi,bi)=h−1{μ2+α⋅Φ(μ1+Xi'β1+bi)},

where h is a known link function, and α and $\mu_{2}$ are unknown parameters. Here, Φ(μ1+Xi'β1+bi)=P(YDji=1|Dji=1,Xi,bi) is the subject-specific sensitivity of the diagnostic endpoint. For the MarginProbe system, for instance, $Y_{Ti}$ represents the re-excision rate, which is a binary endpoint, and can be characterized by a random-effects logistic regression model

log{Prob(YTi=1|Xi,bi)1−Prob(YTi=1|Xi,bi)}=μ2+Xi'β2+α⋅Φ(μ1+Xi'β1+bi).

All models given above together constitute the joint models for the co-primary endpoints $Y_{Ti}$ and $Y_{Di}$. From the joint models, we can derive the expected value of $Y_{Ti}$ as a function of the sensitivity of $Y_{D_{j}i}$ and consequently estimate the expected value of $Y_{Ti}$ when the sensitivity of $Y_{D_{j}i}$ is fixed at 0.5. With the model for the prognostic endpoint, we basically assume the expected value of the prognostic endpoint is a differentiable monotone function of the device's sensitivity. This comply with the conditions for the diagnostic devices that are designed to help to improve the surgical outcomes, such as the MarginProbe system. It can be modified in case it is not reasonable. By adopting the joint modeling approach in the analysis of a non-ND study, we are able to evaluate the performance of the investigational device through the estimated coefficient α, although the direct treated-to-ND effect and the direct treated-to-SOC effect cannot be estimated. For instance, a negative estimate of α indicates that the risk that observes the prognostic endpoint to be 1 (i.e., the re-excision rate in evaluating the MarginProbe system) decreases as the subject-specific sensitivity increases. The treated-to-SOC effect can be estimated by calculating the difference of average values of the prognostic endpoint from the two arms as usual.

## 5 A synthetic clinical example

Conventional statistical analysis approaches for the RCTs can be adopted to analyze the data collected from ND studies. In this section, we present a synthetic non-ND study, in which synthetic data were simulated and analyzed by the joint modeling approach. The synthetic data were simulated according to the combined study results from the three major clinical trials (the MAST [2], the US Pivotal [5], and the German multicenter study [6]) that investigated the MarginProbe system. These studies revealed a 70%–100% sensitivity and 70–87% specificity for the system to detect cancerous breast cancer tissues, resulting in re-excision rates from 5.6% to 17%. The performance of the device mainly depended on size of cancerous tissues ([5]) and other unobserved factors. Combining these empirical outcomes from the clinical trials, we simulated study data for a synthetic non-ND trial as follows.

We assumed there were 500 study subjects (i=1,2,…,500) in the SOC + device arm that evaluated the MarginProbe system or a medical device of such a kind. For each subject, we assumed that surfaces on six fixed locations on the main specimen of lumpectomy [12] were tested by the device and therefore there were six diagnostic endpoints in the study ($Y_{D_{j}i},\;j=1,\;2,\ldots ,6$). Joint models for sensitivity, specificity of the device, as well as the re-excision rate, were specified as

$$P\left(Y_{D_{j}i}=0\;\text{|}\;D_{ji}=0,\;\;X_{i},\;\;b_{i}\;\right)=\text{Φ}\left(1.39-0.12X_{i}+\lambda \cdot b_{i}\right)P\left(Y_{D_{j}i}=1\;\text{|}\;D_{ji}=1,\;\;X_{i},\;\;b_{i}\;\right)=\text{Φ}\left(1.55-0.085X_{i}+b_{i}\right)$$

and

$$\log\left\{\frac{Prob\left(Y_{Ti}=1\;|\;X_{i},\;\;b_{i}\right)}{1-Prob\left(Y_{Ti}=1\;|\;X_{i},\;\;b_{i}\right)}\right\}=v_{Ti}=\beta_{0}+\alpha \cdot \text{Φ}\left(1.55-0.085X_{i}+b_{i}\right)\text{,}$$

in which Φ is the probit link function, some of the regression coefficients were fixed as shown above, the baseline covariate $X_{i}$ (representing the lumpectomy specimen volume in a clinical trial for evaluating the MarginProbe system) was randomly sampled from a normal distribution with a mean of 6 and standard deviation of 1, the true disease status $D_{ji}$ was randomly sampled from a Bernoulli distribution with a 0.5 probability of success, the random effect $b_{i}$ was random sampled from a normal distribution with a mean of 0 and standard deviation of 0.05, and the scaling parameter λ was specified to be 1.5. We considered three α values: (1) α=0, indicating that use of the device does not influence the re-excision rate; (2) α=−2, indicating the re-excision rate moderately decreases as the subject-specified sensitivity increases; and (3) α=−4, indicating the re-excision rate largely decreases as the subject-specified sensitivity increases. Correspondingly, $\beta_{0}$ was set at −2.2, −0.5, and 1.2, respectively. This setting ensured an average subject-specified sensitivity of 0.85, an average subject-specified specificity of 0.75, and the subject-specified re-excision rates close to 0.1.

**Fig. 2:**
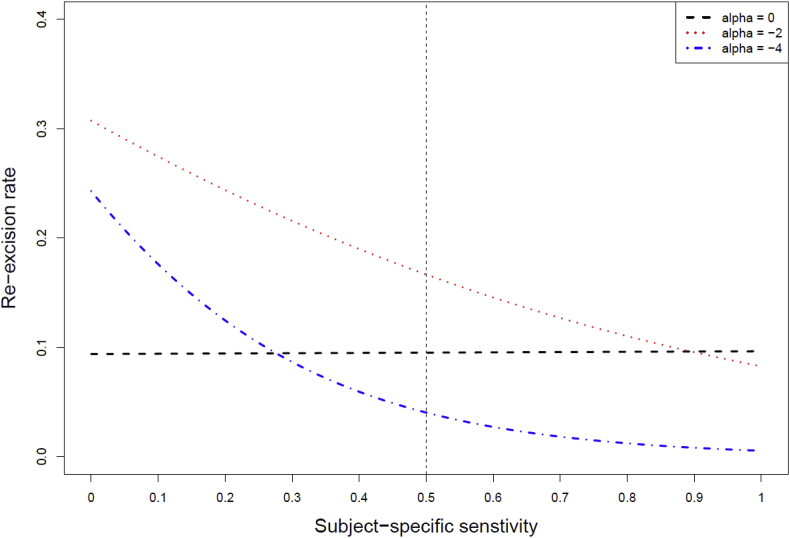
Change of the estimated subjective-specific re-excision rates with the estimated subjective-specific sensitivities for α=0,−2, and −4.

The data from the synthetic ND study (the SOC + device arm) were fitted by the joint models introduced in Section 4 using the NLMIXED procedure in SAS. Table 1 shows estimates of the unknown parameters when α=0,−2, and −4. We observed that all estimates of the unknown parameters were close to their true values with small standard errors, although the variance component estimates for the random effect varied among different models. Fig. 2 demonstrates the change of the estimated subjective-specific re-excision rates with the estimated subjective-specific sensitivities for α=0,−2, and −4.

**Table 1:** Estimates and standard errors (in parentheses) of the unknown parameters when α=0,−2, and −4 in the synthetic clinical study.

Parameter	α=0	α=−2	α=−4
$\mu_{1}$	1.395 (0.012)	1.456 (0.014)	1.519 (0.005)
$\beta_{1}$	−0.065 (0.002)	−0.074 (0.002)	−0.076 (0.007)
$\mu_{0}$	1.695 (0.010)	1.529 (0.021)	1.445 (0.007)
$\beta_{0}$	−0.166 (0.002)	−0.134 (0.004)	−0.070 (0.009)
$\mu_{2}$	−2.267 (0.006)	−0.812 (0.007)	1.137 (0.021)
α	0.030 (0.012)	−1.594 (0.009)	−4.062 (0.026)
σ	0.068 (0.005)	0.141 (0.008)	0.518 (0.047)
λ	1.763 (0.084)	1.630 (0.027)	1.502 (0.012)

## 6 Discussion

The concept of ND proposed in this article is expected to be a game-changer in evaluating the effectiveness and safety of diagnostic medical devices with direct therapeutic purposes. When the ND study design and analysis are thoroughly planned and well-executed, they can be a powerful tool to evaluate breast cancer surgical devices, such as the MarginProbe, or other medical devices with similar functions. If the ND study design is adopted, awkward situations can be avoided, like the FDA approving such a device for breast cancer surgery but admitting to the public that it did not know about the device's true effectiveness because the study was biased [12]. The joint modeling approach proposed herein can help both the sponsors and the FDA review team evaluate the true effectiveness of the device, even when the ND design is not feasible. The direct regulatory impact from adopting the proposed concept and statistical analysis approach will be significant in evaluating the surgical devices that offer a clinically meaningful advantage in providing an intraoperative indication of the margin status as an adjunct to the SOC during breast-conserving surgery and lumpectomy procedures for breast carcinoma.

## 7 Conclusion

In this article, we promote a new study design for medical device clinical trials, named “ND design” for evaluating the effectiveness and safety of new investigational diagnostic medical devices with direct therapeutic functions. We proposed and discussed a joint modeling approach for statistical analysis of the non-ND study when the ND design is not feasible in practice. The proposed methods can help the sponsors and FDA regulators to efficiently evaluate the effectiveness and safety of new investigational diagnostic medical devices with direct therapeutic functions.

## Funding

Dr. Bo Zhang's research was partially supported by the National Institutes of Health grant U24 AA026968 and the University of Massachusetts Center for Clinical and Translational Science grant UL1TR001453, TL1TR01454, and KL2TR01455.

## References

[1] Karni T., Pappo I., Sandbank J., Lavon O., Kent V., Spector R., Morgenstern S., Lelcuk S. (2007 Oct 1). A device for real-time, intraoperative margin assessment in breast-conservation surgery. Am. J. Surg..

[2] Allweis T.M., Kaufman Z., Lelcuk S., Pappo I., Karni T., Schneebaum S., Spector R., Schindel A., Hershko D., Zilberman M., Sayfan J. (2008 Oct 1). A prospective, randomized, controlled, multicenter study of a real-time, intraoperative probe for positive margin detection in breast-conserving surgery. Am. J. Surg..

[3] Thill M. (2013 May 1). MarginProbe®: intraoperative margin assessment during breast conserving surgery by using radiofrequency spectroscopy. Expert Rev. Med. Devices.

[4] Rivera R.J., Holmes D.R., Tafra L. (2012). Analysis of the impact of intraoperative margin assessment with adjunctive use of MarginProbe versus standard of care on tissue volume removed. Int. J. Surg. Oncol..

[5] Schnabel F., Boolbol S.K., Gittleman M., Karni T., Tafra L., Feldman S., Police A., Friedman N.B., Karlan S., Holmes D., Willey S.C. (2014 May 1). A randomized prospective study of lumpectomy margin assessment with use of marginprobe in patients with nonpalpable breast malignancies. Ann. Surg. Oncol..

[6] Thill M., Dittmer C., Baumann K., Friedrichs K., Blohmer J.U. (2014 Feb 1). MarginProbe® - Final results of the German post-market study in breast conserving surgery of ductal carcinoma in situ. Breast.

[7] Thill M., Röder K., Diedrich K., Dittmer C. (2011 Dec 1). Intraoperative assessment of surgical margins during breast conserving surgery of ductal carcinoma in situ by use of radiofrequency spectroscopy. Breast.

[8] Sebastian M., Akbari S., Anglin B., Lin E.H., Police A.M. (2015 Dec 1). The impact of use of an intraoperative margin assessment device on re-excision rates. Springerplus.

[9] Blohmer J.U., Tanko J., Kueper J., Groß J., Völker R., Machleidt A. (2016 Aug 1). MarginProbe© reduces the rate of re-excision following breast conserving surgery for breast cancer. Arch. Gynecol. Obstet..

[10] Pappo I., Spector R., Schindel A., Morgenstern S., Sandbank J., Leider L.T., Schneebaum S., Lelcuk S., Karni T. (2010 May 15). Diagnostic performance of a novel device for real-time margin assessment in lumpectomy specimens. J. Surg. Res..

[11] Thill M., Baumann K., Barinoff J. (2014 Jul). Intraoperative assessment of margins in breast conservative surgery - still in use?. J. Surg. Oncol..

[12] Food U.S., Administration D. (2013). Summary of Safety and Effectiveness Data: MarginProbe®System. 2012.

[13] Chen Z., Zhang B., Albert P.S. (2011 Jul 10). A joint modeling approach to data with informative cluster size: robustness to the cluster size model. Stat. Med..

[14] Zhang B., Liu W., Zhang Z., Qu Y., Chen Z., Albert P.S. (2017 Aug). Modeling of correlated data with informative cluster sizes: an evaluation of joint modeling and within-cluster resampling approaches. Stat. Methods Med. Res..

